# Cholecystectomy Is a Risk Factor for Microscopic Colitis: A Nationwide Population-based Matched Case Control Study

**DOI:** 10.1016/j.cgh.2024.12.032

**Published:** 2025-03-11

**Authors:** David Bergman, Fahim Ebrahimi, Jiangwei Sun, John Maret-Ouda, Björn Lindkvist, Anne Peery, Jonas F. Ludvigsson

**Affiliations:** 1 Department of Medical Epidemiology and Biostatistics, Karolinska Institutet, Stockholm, Sweden; 2 Department of Gastroenterology and Hepatology, University Digestive Health Care Center Basel – Clarunis, Basel, Switzerland; 3 Department of Internal Medicine, Sahlgrenska University Hospital, Gothenburg, Sweden; 4 Department of Medicine, University of North Carolina, Chapel Hill, North Carolina; 5 Department of Pediatrics, Orebro University Hospital, Orebro, Sweden; 6 Department of Medicine, Columbia University College of Physicians and Surgeons, New York, New York

**Keywords:** Bile Flow, Case-control Study, Cholecystectomy, Epidemiology, Microscopic Colitis, Nationwide

## Abstract

**BACKGROUND & AIMS:**

Studies have linked bile acid malabsorption and microscopic colitis (MC), with some patients with MC responding to treatment with bile acid sequestrants. However, the literature on cholecystectomy as a risk factor for MC is inconclusive. Therefore, we investigated the relationship between cholecystectomy and MC on a nationwide scale to provide more definitive insights.

**METHODS:**

We conducted a nationwide matched case-control study involving 13,554 patients diagnosed with MC between 1981 and 2017 in Sweden who were matched to 64,886 controls. Data on MC were obtained from Swedish pathology registers, and controls were randomly selected from the general population and matched according to birth year, sex, county of residence, and calendar year. Moreover, MC-free full siblings to patients with MC were identified. Information on cholecystectomy was collected from the Swedish National Patient Register. Adjusted odds ratios (aORs) were calculated using multivariable-adjusted conditional logistic regression.

**RESULTS:**

The median age at diagnosis was 63.5 years (interquartile range [IQR], 51.0–73.4 years), and 72.3% of MC patients were women. Among patients with MC, 342 (2.5%) had undergone a cholecystectomy before diagnosis, compared with 687 (1.1%) in the control group. This yielded an aOR of 2.36 (95% confidence interval [CI], 2.07–2.69) for earlier cholecystectomy in patients with MC. The corresponding aORs for collagenous colitis and lymphocytic colitis were 1.87 (95% CI, 1.48–2.36) and 2.65 (95% CI, 2.26–3.12), respectively. When compared with siblings, the aOR was 1.49 (95% CI, 1.21–1.85).

**CONCLUSIONS:**

Cholecystectomy is associated with an increased risk of subsequent MC. These findings have implications for surgeons and general practitioners and underscore the need for further research into the underlying association between bile acid and MC.

## Graphical Abstract

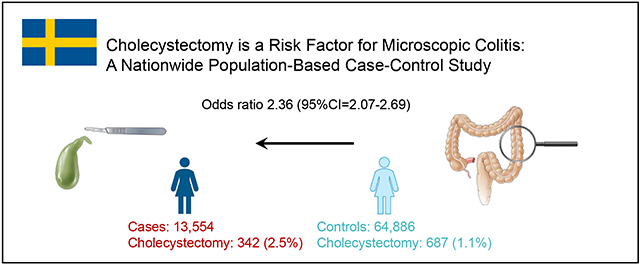

Microscopic colitis (MC) is an inflammatory condition of the large intestine. The term MC is a unifying concept for 2 subtypes distinguished by their histopathologic presentation, collagenous colitis (CC) and lymphocytic colitis (LC).^1^ An estimated 0.9% of women and 0.4% men will develop MC.^2^ The most prominent symptoms are frequent, non-bloody watery diarrhea, weight loss, and abdominal pain.^3^ Some patients may also experience nocturnal diarrhea, urgency, and fecal incontinence.^4^ The severity of symptoms may, however, differ substantially between patients.

There is a known overlap between MC and bile salt malabsorption,^5–7^ with up to 60% of patients with LC having a 75 Se-labelled homocholic acid-taurine (75 SeHCAT) test indicative of bile salt malabsorption. Moreover, some 20% of patients with MC have been found to have a clinical response to bile acid sequestrants.^8^ Recent evidence from a Portuguese case-control study^9^ indicates that the farnesoid-x receptor (FXR) plays an important part in the association between MC and bile flow and that patients with MC have a lower expression of this receptor. The downstream effect of a decreased FXR activity is a decreased concentration of fibroblast growth factor 19 (FGF-19) in the enterohepatic circulation.^9^ As FGF-19 provides negative feedback on the production of bile acids in the liver, patients with future MC may have a decreased ability to hamper production of bile acids in the liver and thus be more sensitive to procedures resulting in an altered bile flow.

In addition to the functional disruption (ie, idiopathic bile salt malabsorption due to a dysregulated recycling of bile acids or an excess bile salt production) associated with bile salt malabsorption, normal bile flow and enterohepatic circulation may also be altered after surgical procedures.

As cholecystectomies leads to the loss of the bile reservoir, these procedures are inherently capable of affecting bile flow.

To date, the association between cholecystectomy and MC has only been explored in a few studies. Three United States studies,^10–12^ based on some 100 patients with MC per study, have reported conflicting results (2 studies found no significant associations with previous cholecystectomy, whereas one study found previous cholecystectomy to be less common among patients with MC compared with controls with chronic diarrhea. Although well-conducted, these studies were based on small populations from a limited catchment area; one county [n = 130]^10^ or one center [n = 93, only published as an abstract],^11^ and n = 110^12^). To the best of our knowledge, there are no studies investigating this potential association on a larger scale.

Hence, we aimed to leverage a large, nationwide cohort to revisit the question of whether cholecystectomy is a risk factor for MC.

## Materials and Methods

### Setting

Health care in Sweden is almost exclusively funded by the public sector and designed to provide all citizens with equal access to medical services. The information used for this study was sourced from nationwide health care registers in Sweden, along with government-maintained databases on the Swedish population. At birth or immigration, all Swedish residents are assigned a unique personal identity number,^13^ facilitating a comprehensive collection of relevant data on study participants from diverse sources, resulting in minimal data gaps.

### Data Sources

Information regarding our study’s exposures was gathered from the Swedish National Patient Register (NPR),^14^ which compiles data on diagnoses and medical procedures using International Classification of Diseases (ICD), OPKOD, or Nordic Medico-Statistical Committee (NOMESCO) codes. The NPR achieved nationwide coverage in 1987 and has, since 2001, also incorporated data from specialized outpatient care facilities.

Details concerning prescribed medications were obtained from the Prescribed Drug Register (PDR).^15^ Since July 1, 2005, this register has maintained nearly complete coverage of dispensed medications across Sweden.

Matched controls were identified from the Total Population Register,^16^ which contains records on personal identity numbers, vital status, migration, and residency of all Swedish citizens dating back to 1968. Siblings of patients with MC were identified through linkage to the Multigeneration Register,^16^ a subset of the Total Population Register. This register stores information on all Swedish citizens born from 1932 to the present day, along with their siblings and biological parents. Only siblings without a biopsy indicating MC were included.

The ESPRESSO (Epidemiology Strengthened by histoPathology Reports in Sweden) study^17^ has gathered data on all gastrointestinal (GI) biopsies taken in Sweden from 1965 to 2017. Consequently, this data source enabled the identification of individuals with colonic biopsies consistent with MC. Subsequently, up to 5 controls per MC patient were randomly selected from the general population to establish a matching set. These controls were matched based on sex, birth year, calendar period, and county of residence at the time of MC diagnosis.

### Definition of Cholecystectomy

Our exposure, cholecystectomy, was comprised of several pre-defined procedures (Supplementary Table 1), occurring before the matching date. The relevant surgical procedures were identified using surgical procedure codes according to the OPKOD and NOMESCO systems. As MC was not routinely diagnosed in Sweden before the 1990s, we defined exposure as having occurred within 10 years before index date. This was done to avoid the risk of time-lag bias, which stems from us having data on cholecystectomies from 1964 while the first record of a patient with MC is from 1981. Hence, not including a time-limit would risk misclassifying exposed patients as not having had the outcome.

### Ascertainment of MC

Sweden hosts 28 regional pathology registers, all employing the Systematised Nomenclature of Medicine (SNOMED) system to store information on biopsy morphology. In our current study, MC was defined using SNOMED codes for CC (M40600) or LC (M47170) registered between 1981 and 2017. In a prior investigation, where patient charts with biopsies indicative of MC were randomly selected and reviewed, we observed a positive predictive value (PPV) of 95%. Additionally, 96% of patients reported diarrhea before diagnosis, affirming the accuracy of our definition in identifying clinically active disease.^18^

### Covariates

Baseline characteristics such as sex, age, county of residence, and country of birth (Nordic [Sweden, Denmark, Norway, Finland, Iceland] or other) for all individuals were obtained from the Total Population Register.^16^

Educational attainment served as proxy for socioeconomic status, with educational levels categorized as compulsory school (≤9 years), upper secondary school (10–12 years), or college (≥13 years) (Supplementary Table 1). This information was collected from the longitudinal integrated database for health insurance and labor market studies.^19^ In cases where data on the level of education was unavailable, the highest level of education of the parents was used. For instances where information on the parents’ education was also missing, the study participants were placed in a “missing” category.

Given the absence of a validated method to assess disease severity in MC, we used the amount of dispensed budesonide (Anatomical Therapeutic Chemical [ATC] code A07EA06 with doses 3 or 9 mg) from 7 days before MC diagnosis and during the first year after diagnosis as the basis for classifying disease intensity (retrievals of budesonide 9 mg were recalculated to 3 pills × 3 mg). Disease intensity categories were quiescent (no retrievals of budesonide), sustained remission (1–250 pills), relapsing (251–500 pills), and chronic (>500 pills). The thresholds were chosen based on a typical 3-month taper in Sweden (during the study period) that would require the equivalent of 168 budesonide pills at 3 mg. Thus, a patient characterized as being in remission would have had 1 and less than 2 full dispensations, relapsing would correspond to more than 1 but less than 3, and chronic would correspond to 3 or more dispensations. Data on dispensed budesonide was gathered from the PDR.^15^ Comprehensive information on definition of comorbidities is included in Supplementary Table 1.

### Exclusion Criteria

**Figure 1.**
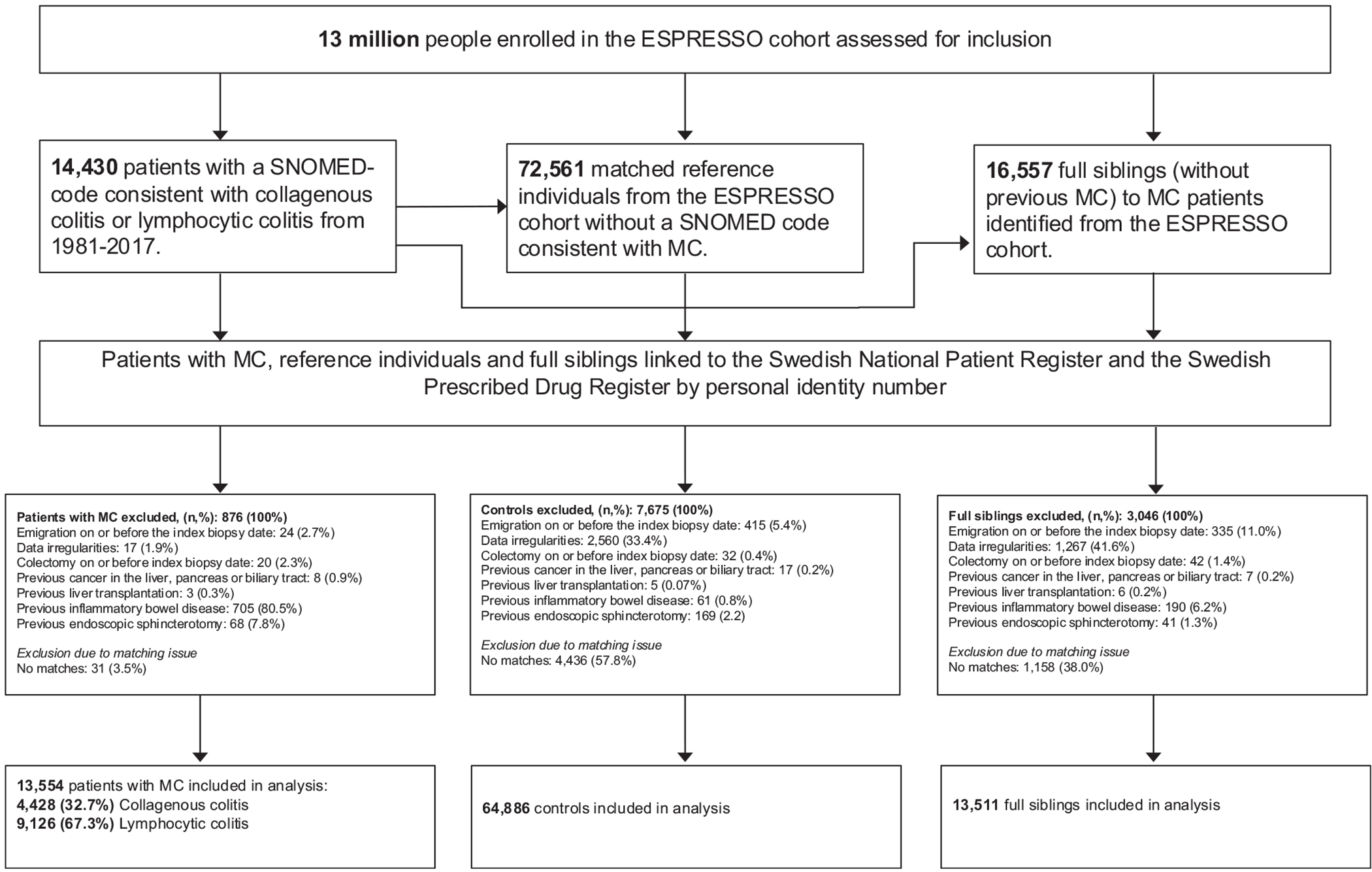
Flowchart of inclusion of patients with biopsy-confirmed microscopic colitis in the ESPRESSO histopathology cohort, siblings, and matched general population controls from the Swedish Total Population Register 1981–2017.

Figure 1 illustrates the study exclusions. Uniform exclusion criteria were applied to all participants. Specifically, individuals with data irregularities related to emigration or death, or who underwent colectomy before the matching date, were excluded. Additionally, patients with a history of endoscopic sphincterotomy, liver transplantation, diagnosis of liver cancer, pancreas cancer, or biliary tract cancers, as well as those with inflammatory bowel disease (IBD),^20^ were excluded.

### Sensitivity Analyses

First, to control for the impact of sphincterotomy, we added endoscopic sphincterotomy to our exposure definition (for definitions, see Supplementary Table 1). Second, altered bile flow following cholecystectomy could elevate bile acid levels in the colon of patients with MC, potentially exacerbating disease symptoms. Hence, we hypothesized an association between cholecystectomy and more severe MC. To test this hypothesis, we varied the outcome based on the disease intensity definitions outlined in the covariates section. As these analyses required data from the NPR, only patients with an index/MC diagnosis date after July 1, 2005, were included. Third, to control for surveillance bias, we excluded all study participants with a follow-up of less than 1 year. Fourth, to control for the impact of smoking, our model was additionally adjusted for chronic obstructive pulmonary disease (COPD), as a proxy for heavy smoking (see Supplementary Table 1 for definition). Finally, to assess the effect of familial confounding and coaggregation, full siblings, regardless of sex, were used as controls.

## Patient and Public Involvement

No patient participated in the planning or design of this study.

### Statistical Analysis

We examined the frequency of previous cholecystectomy in patients with MC compared with matched controls. Odds ratios (ORs) and 95% confidence intervals (CIs) were calculated using conditional logistic regression.

The analysis was conditioned on our matching variables (birth year, sex, county of residence, and year of biopsy) and additionally adjusted for educational attainment to prevent confounding by these factors.

Subgroup analyses were conducted based on sex, age at the index date (<50 or ≥50 years), calendar year at matching date (<1990, 1990–2000, 2001–2010, 2011–2017), country of birth (Nordic or other), and educational attainment (≤9, 10–12, ≥13 years, or missing) using the same model.

In the sibling-controlled analysis, comparisons were made exclusively between patients with MC and their MC-free, full siblings.

Statistical significance of effect modification was tested by introducing an interaction term that included MC status and each of the above strata into the main model.

A *P*-value of < .05 was considered statistically significant.

All statistical analyses used Stata/IC 17.0 for Mac (StataCorp).

### Ethical Approval

This study was approved by the Regional Ethics Committee, Stockholm, Sweden (Protocol no 2014/1287-31/4, 2018/972-32 and 2022-05774-02). Because the study was strictly register-based, informed consent was not required.^21^

## Results

Between 1981 and 2017, we identified 13,554 patients with incident MC. These cases were matched with 64,886 controls from the general population (Table 1). Additionally, 13,511 full siblings without MC were identified. As anticipated, the majority (72.3%) of patients with MC were women. The median age at MC diagnosis was 63.5 years (interquartile range [IQR], 51.0–73.4). A significant portion (94%) of patients with MC were born in a Nordic country, and some 30% had attained a university or college education (equivalent to 13 years of schooling or more).

### Cholecystectomy Preceding MC

In total, 1029 patients had a record of a cholecystectomy. Of these, 342 occurred in patients who were subsequently (within 10 years) diagnosed with MC (representing 2.5% of all patients with MC), whereas 1.1% of controls (n = 687) had a prior record of such procedures. The median age at the time of cholecystectomy was nearly identical for both cases (57.9 years; IQR, 48.3–66.5 years) and controls (57.7 years; IQR, 48.0–65.4 years). Median time from cholecystectomy to MC was 4.5 years (IQR, 2.3–7.4 years).

When conditioning on the matching variables (birth year, sex, county, calendar year), and adjusting for educational attainment (as a proxy for socioeconomic status), we obtained an adjusted odds ratio (aOR) of 2.36 (95% CI, 2.07–2.69) (Table 2) for cholecystectomy in patients with MC. Stratifying by sex, the aORs were comparable, albeit with a slightly wider CI for males, reflecting the female predominance in MC. Analyses based on age at the matching date (younger or older than 50 years) yielded similar estimates. We also stratified by calendar period at matching date. Due to MC not being routinely diagnosed in Sweden before the mid-1990s, the estimate for the initial calendar period (≤1990) was uninformative, whereas those for subsequent periods (1991–2000, 2001–2010, and 2011–2017) decreased with more recent calendar periods (*P* for trend = .001), perhaps indicating a more marked influence of surveillance bias in the earlier calendar periods.

Stratification by subtype revealed a higher aOR for LC (2.65; 95% CI, 2.26–3.12) compared with CC (1.87; 95% CI, 1.48–2.36; *P*_heterogeneity_ = .016).

### Secondary and Sensitivity Analyses

**Figure 2.**
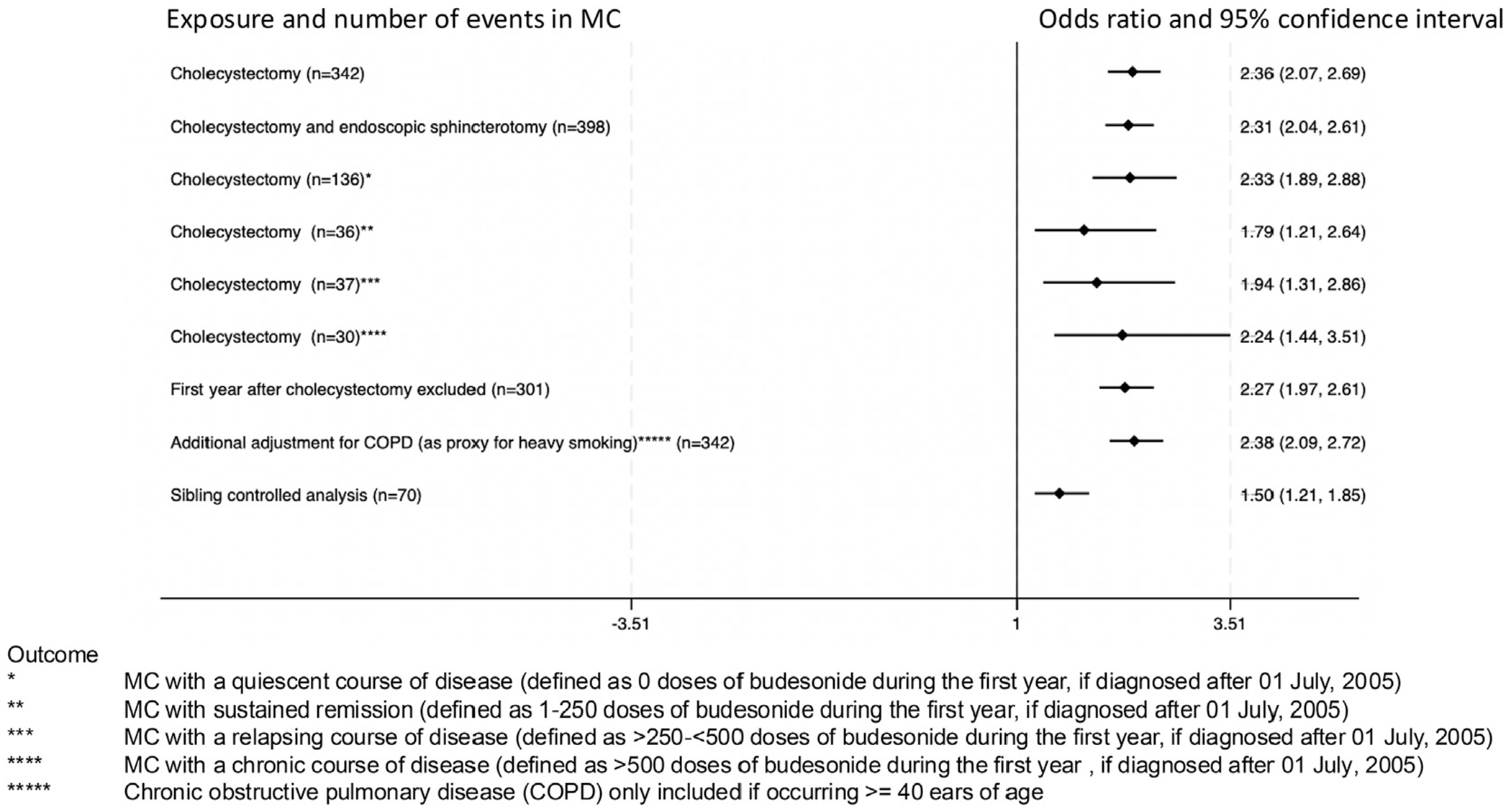
Forest plot depicting ORs for earlier cholecystectomy among patients with MC. Bile flow-altering procedures are defined as cholecystectomy, cholecystectomy + choledochotomy, cholecystectomy + choledocholithotomy, laparoscopic cholecystectomy without sphincterotomy, cholecystectomy + choledocholithotomy + sphincterotomy, and other related procedures.

Comprehensive results of our secondary analyses are presented in Figure 2. Briefly, these analyses corroborated our main findings. No evidence was found for cholecystectomy influencing disease intensity in MC (*P* for heterogeneity = .32). Moreover, diagnostic timing of MC in relationship to previous cholecystectomy did not have an impact on disease intensity as median time in years from exposure to outcome was comparative across all levels of disease intensity (quiescent: 4.6; IQR, 1.8–7.5; sustained remission: 3.7; IQR, 2.4–7.4; relapsing: 5.8; IQR, 3.3–7.4; and chronic: 4.2; IQR, 2.6–6.2).

Furthermore, dispensed cholestyramine did not have a substantial impact on the use of budesonide during the first year after diagnosis, as the proportion of patients treated with cholestyramine varied between 4% and 5% across all activity groups, with no apparent trend. When stratifying by prior cholecystectomy status, an additional pattern emerged: among patients with MC and a previous cholecystectomy, 26% had a recorded dispensation of cholestyramine (after the cholecystectomy but before MC diagnosis), compared with 18% in patients without a prior cholecystectomy.

When using siblings as controls, we obtained a lower, but still significant aOR (1.31; 95% CI, 1.07–1.61).

## Discussion

In this nationwide population-based matched case-control study of 13,554 patients with MC, we found that patients having undergone a cholecystectomy were at a more than doubled risk of later MC. The association was robust across several sensitivity analyses. However, preceding cholecystectomy had no meaningful impact on MC disease severity as measured by budesonide treatment. The positive association with cholecystectomy was also observed in the sibling-controlled analysis, albeit with a slightly lower OR, indicating that our findings are not entirely explained by shared genetics or early environmental factors. The lack of association between cholecystectomy and disease intensity aligns with previous observations that histologic inflammation does not correlate with symptom burden in MC.^22^ Similarly, we found no association between a history of cholecystectomy—which could potentially increase bile flow and thereby lead to increased inflammation^23^—and budesonide use, a proxy for symptom severity in MC. These results suggest that the potentially increased bile acid-related inflammation resulting from a cholecystectomy might not translate to a higher symptom burden, underscoring the complexity of symptom mechanisms in MC.

### A Comparison With the Literature

The association between cholecystectomy and MC has garnered limited attention in the existing literature. Recent studies have provided notable insights, yet with diverging and inconsistent estimates,^10–12^ perhaps due to small sample sizes. However, one of these studies^12^ used patients referred for colonoscopy due to chronic diarrhea as controls and found prior cholecystectomy to occur less frequently in cases (MC) compared with controls. In contrast with diarrhea controls, we used the general population to assess the association between cholecystectomy and MC.

Unfortunately, as we do not have data on the indication for referrals, we were unable to conduct a similar analysis. Our current findings align with those from our earlier study on MC and future acute pancreatitis^24^ (AP), in which we found a significant association between MC and non-gallstone-related AP but no association with gallstone-related AP. A potential mechanism could be that altered bile flow after cholecystectomy, particularly in patients with lower expression of the FXR receptor (which normally helps mitigate mucosal inflammation in the colon), may trigger MC. This, in turn, may activate the inflammatory pathways implicated in the association between MC and non-gallstone-related AP.

Although the link between bile salt malabsorption and MC has been explored,^5–7^ comprehensive epidemiologic investigations into the impact of cholecystectomy on MC development are lacking.

### Biologic Mechanisms

Our findings are biologically plausible. As mentioned in the introduction, approximately 20% of patients with MC exhibit a clinical response to bile acid sequestrants.^8^ Additionally, studies have reported lower expression of the FXR receptor in the colon of patients with MC.^9^ It is not clear whether this lower expression occurs as a result of a negative feedback loop initiated by bile acid malabsorption or if it is a side effect of the inflammatory changes caused by increased levels of bile acids in the colon. However, given that the FXR receptor plays a crucial role in the enterohepatic feedback loop that regulates bile acid production in the liver, our results may partly be explained by an increased sensitivity for alterations in bile flow among individuals with a susceptibility for MC. Moreover, elevated concentrations of bile acids in the intestines can lead to mucosal inflammation and diarrhea, bringing the patient to clinical attention. Finally, experimental studies have demonstrated that even low levels of bile acids can increase bacterial uptake in colonic biopsies,^25^ highlighting the potential role of bile acids in the dysfunction of the mucosal barrier, a hallmark of MC and implicated in the pathogenesis of the disease. Animal models have also reported that bile acids may induce histologic changes resembling LC,^23^ which may explain our finding of a somewhat higher estimate for LC.

### Strengths and Weaknesses

Our study possesses several strengths. First, the nationwide coverage of the ESPRESSO study^16^ ensures minimal impact of selection bias. This extensive coverage, combined with the size of our cohort, enabled precise computations across various strata. Additionally, our previous validation work^15^ lends credibility to our definition of MC. Moreover, the use of personal identity numbers^21^ facilitated linkage to multiple population-based registers, allowing for the construction of a large and comprehensive cohort of cases and controls with long follow-up. Lastly, our ability to identify full siblings provided a unique opportunity to address intrafamilial confounding.

However, there are also limitations to consider. Our reliance on data from Swedish registers means we lack information on potentially confounding lifestyle factors such as body mass index (BMI), eating habits and clinical parameters such as the Hjortswang criteria to assess disease activity in MC. The latter limitation was the primary reason for the budesonide-based activity index used in the analysis. However, BMI appears unlikely to be of major importance in this context, as MC has been linked to a low BMI,^25^ whereas a higher BMI is a major risk factor for acute cholecystitis, a leading cause for cholecystectomy. Hence, the lack of BMI in our model may have underestimated the true risk of MC among patients with cholecystectomy. Additionally, our inability to conduct a cohort study due to data availability (the ESPRESSO cohort is based on patients having had a biopsy from the GI tract, and in most cases, a biopsy is not taken during a cholecystectomy) prevented us from calculating absolute risks. It is also important to note that only a small proportion of patients with MC had a previous record of cholecystectomy. Finally, as our study is based on data from the Swedish population, the external validity may be limited, and our results may not be directly applicable to populations with different lifestyle factors, comorbidities, and ethnicities.

Our findings underscore the need for further research to explore the mechanisms explaining a potential association between cholecystectomy and later MC. In particular, studies investigating the specific role of bile acid sequestrants in the treatment of MC could help refine our approach to management. Additionally, a deeper understanding of bile acid signaling, the FXR receptor, and its influence on gut inflammation in MC would be a valuable contribution to the literature.

In conclusion, cholecystectomy poses a risk factor for MC. Our findings have clinical significance, emphasizing the importance of advising patients who have undergone cholecystectomy to seek medical attention in case of diarrhea onset. Also, clinicians should consider MC in cases where diarrhea post-cholecystectomy does not respond to bile acid sequestrants. Additionally, our findings contribute to scientific knowledge by providing motivation for further mechanistic research.

## Supplementary Material

1

Note: To access the supplementary material accompanying this article, visit the online version of *Clinical Gastroenterology and Hepatology* at www.cghjournal.org, and at https://doi.org/10.1016/j.cgh.2024.12.032.

## Figures and Tables

**Table 1.** Summary Statistics for Patients With MC and Matched Controls

	Controls	MC	CC	LC
Total	64,886 (100.00)	13,554 (100.00)	4428 (100.00)	9126 (100.00)

Male	17,879 (27.6)	3756 (27.7)	1019 (23.0)	2737 (30.0)

Female	46,987 (72.4)	9798 (72.3)	3409 (77.0)	6389 (70.0)

Age, years				
Mean (SD)	60.4 (16.7)	61.0 (16.7)	63.9 (15.0)	59.5 (17.3)
Median (IQR)	62.9 (50.5-72.7)	63.5 (51.0-73.4)	65.6 (55.0-74.9)	62.3 (48.9-72.5)
<50	15,766 (24.3)	3191 (23.5)	758 (17.1)	2433 (26.7)
≥50	49,100 (75.7)	10,363 (76.5)	3670 (82.9)	6693 (73.3)

Year of matching date				
≤1990	20 (0.03)	4 (0.03)	0 (0)	4 (0.04)
1991-2000	4,269 (6.6)	878 (6.5)	274 (6.2)	604 (6.6)
2001-2010	34,137 (52.6)	7119 (52.5)	2418 (54.6)	4701 (51.5)
2011-2017	26,440 (40.8)	5553 (41.0)	1736 (39.2)	3817 (41.8)

Cholecystectomy prior to matching date Cholecystectomy^a^	687 (1.1)	342 (2.5)	103 (2.3)	239 (2.6)

Country of birth				
Nordic	57,467 (88.6)	12,715 (93.8)	4242 (95.8)	8473 (92.8)
Other	7399 (11.4)	839 (6.2)	186 (4.2)	653 (7.2)

Education				
Compulsory school (≤9 years)	18,131 (28.0)	3577 (26.4)	1337 (30.2)	2240 (24.6)
Upper secondary school (10–12 years)	25,856 (39.9)	5524 (40.8)	1836 (41.5)	3688 (40.4)
College or university (≥13 years)	18,624 (28.7)	4091 (30.2)	1152 (26.0)	2939 (32.2)
Missing	2255 (3.5)	362 (2.7)	103 (2.3)	259 (2.8)

Note: Data are presented as number (%).

CC, collagenous colitis; IQR, interquartile range; LC, lymphocytic colitis; MC, microscopic colitis; SD, standard deviation.

a Defined as cholecystectomy, cholecystectomy + choledochotomy, cholecystectomy + choledocholithectomy, laparoscopic cholecystectomy without sphincterotomy, cholecystectomy + choledocholithectomy + sphincterotomy, other related procedures. For definitions of exposures, see Supplementary Table 1 for relevant International Classification of Disease (ICD) codes.

**Table 2.** ORs (cholecystectomy^a^) for Patients With MC Diagnosed in Sweden Compared With Matched Controls

	Number of events (cholecystectomy preceding matching date)		MC	CC	LC
	Controls (n = 64,886)	Patients with MC (n = 13,554)	OR (95% CI)	OR (95% CI)	OR (95% CI)
Total	687	342	2.36 (2.07–2.69)	1.87 (1.48–2.36)	2.65 (2.26–3.12)

Sex					
Males	110	61	2.56 (1.86–3.53)	1.78 (0.98–3.23)	3.02 (2.06–4.42)
Females	577	281	2.32 (2.00–2.68)	1.89 (1.47–2.43)	2.58 (2.16–3.08)

Age at start follow-up, *years*					
<50	144	71	2.46 (1.84–3.29)	2.65 (1.53–4.61)	2.39 (1.70–3.36)
≥50	543	271	2.33 (2.01–2.70)	1.75 (1.36–2.27)	2.72 (2.27–3.27)

Year of biopsy					
≤1990	1	0	NA (NA–NA)^b^	NA (NA–NA)^b^	NA (NA–NA)^b^
1991–2000	38	29	3.84 (2.35–6.27)	2.44 (0.82–7.30)	4.36 (2.50–7.60)
2001–2010	353	198	2.67 (2.24–3.19)	2.20 (1.63–2.96)	3.00 (2.40–3.75)
2011–2017	295	115	1.82 (1.47–2.27)	1.40 (0.94–2.08)	2.07 (1.59–2.68)

Country of birth					
Nordic	586	316	2.29 (1.99–2.63)	1.80 (1.41–2.29)	2.60.48 (2.19–3.09)
Other	10	10	1.55 (0.56–4.32)	1.87 (0.10–35.2)	1.48 (0.50–4.43)

Education, *years*					
Compulsory school (≤ 9)	69	73	2.26 (1.61–3.19)	1.25 (0.69–2.26)	3.12 (2.03–4.81)
Upper secondary school (10–12)	118	139	2.52 (1.85–3.26)	2.16 (1.40–3.33)	2.74 (1.99–3.78)
College or university (≥13)	65	71	2.23 (1.56–3.18)	1.72 (0.84–3.51)	2.43 (1.61–3.66)
Unknown	1	1	2.24 (0.11–42.9)	NA (NA–NA)^b^	2.23 (0.11–44.9)^b^

Note: Adjusted for age, sex, county, calendar period, and level of education.

CI, confidence interval; CC, collagenous colitis; LC, lymphocytic colitis; MC, microscopic colitis; OR, odds ratio.

a Defined as cholecystectomy, cholecystectomy + choledochotomy, cholecystectomy + choledocholithotomy, laparoscopic cholecystectomy without sphincterotomy, cholecystectomy + choledocholithotomy + sphincterotomy, other related procedures. For definitions of exposures, see Supplementary Table 1 for relevant International Classification of Disease (ICD) codes.

b No ORs could be calculated due to insufficient numbers of events.

## Data Availability

In accordance with Swedish regulations, the data from this study are not publicly available.

## What You Need to Know

### Background

Microscopic colitis (MC) has been associated with bile salt malabsorption, and some patients respond to bile acid sequestrants. However, large-scale studies on the link between cholecystectomy and MC are lacking.

### Findings

Our nationwide case-control study in Sweden revealed that prior cholecystectomy was significantly more common in patients with MC than in controls, with an adjusted hazard ratio of 2.36 (95% confidence interval, 2.07–2.69).

### Implications for patient care

Clinicians should consider colonoscopy and evaluation for MC in patients presenting with diarrhea after cholecystectomy.

## Abbreviations used in this paper:

75 SeHCAT: 75 Se-labelled homocholic acid-taurine
aOR: adjusted odds ratio
AP: acute pancreatitis
ATC: Anatomical Therapeutic Chemical
BMI: body mass index
CC: collagenous colitis
CI: confidence interval
COPD: chronic obstructive pulmonary disease
ESPRESSO: Epidemiology Strengthened by histoPathology Reports in Sweden
FXR: Farnesoid-x receptor
FGF-19: fibroblast growth factor 19
GI: gastrointestinal
IBD: inflammatory bowel disease
ICD: International Classification of Diseases
IQR: interquartile range
LC: lymphocytic colitis
MC: microscopic colitis
NOMESCO: Nordic Medico-Statistical Committee
NPR: National Patient Register
OR: odds ratio
PPV: positive predictive value
PDR: Prescribed Drug Register
SNOMED: Systematised Nomenclature of Medicine
