# Supplementary material for: Cholecystectomy Is a Risk Factor for Microscopic Colitis: A Nationwide Population-based Matched Case Control Study
Source: Clin Gastroenterol Hepatol. Author manuscript; Available in PMC 2026 Jun 10. (PMC13250853; doi:10.1016/j.cgh.2024.12.032)
Supplement: 1 [file NIHMS2173379-supplement-1.pdf]

Supplementary Reference

1. Forss A, Clements M, Bergman D, Roelstraete B, Kaplan GG, Myrelid P, et al. A nationwide cohort study of the incidence of inflammatory bowel disease in Sweden from 1990 to 2014. Aliment Pharmacol Ther 2022;55:691–699.

Supplementary Table 1. Definitions of Exclusion Criteria and Exposure

Exclusion criteria – one code prior to index date required for exclusion.	ICD-7	ICD-9/6th version/OPKOD	ICD-10/7th version/ NOMESCO
Liver cancer	155.0, 155.1, 156		
Pancreatic cancer	157, 195.5		
Biliary tract cancer	155.0-3, 152.0, 155.8-9		
Liver transplantation		520.0-9	Z94.4, JJC, DJ00.5-6, V42H
Colectomy before index date (excluded)		4650<br>4651<br>4652<br>4653<br>4654	JFC40-41<br>JFG53<br>JFG73<br>JFG80<br>JFG86<br>JFG96<br>JGB40<br>JGB50<br>JGB60<br>JGB61
Endoscopic papillotomy or sphincterotomy			JKE02
Inflammatory bowel disease		According to definition in[1]	
**OPKOD/NOMESCO codes defining exposure**	**ICD-7**	**ICD-9/6th version/OPKOD**	**ICD-10/7th version/ NOMESCO**
Cholecystectomy		5350	JKA20
Cholecystectomy + choledochotomy		5351	
Cholecystectomy + choledocholithectomy		5352	
Laparoscopic cholecystectomy without sphincterotomy		5353	JKA21
Cholecystectomy + choledocholithectomy + sphincterotomy		5356	
Cholecystectomy + choledocholithectomy + sphincterotomy		5357	
Other related procedures		5359	
Post cholecystectomy syndrome		576A	K915

ICD, International Classification of Diseases; NOMESCO, Nordic Medico-Statistical Committee.
